# In praise of arrays

**DOI:** 10.1007/s00467-008-0808-z

**Published:** 2009-09-01

**Authors:** Lihua Ying, Minnie Sarwal

**Affiliations:** Department of Pediatrics, Stanford University, G320, 300 Pasteur Drive, Stanford, CA 94305 USA

**Keywords:** Microarray, Gene expression, Transplantation, Rejection, Immunosuppression, Monitoring

## Abstract

Microarray technologies have both fascinated and frustrated the transplant community since their introduction roughly a decade ago. Fascination arose from the possibility offered by the technology to gain a profound insight into the cellular response to immunogenic injury and the potential that this genomic signature would be indicative of the biological mechanism by which that stress was induced. Frustrations have arisen primarily from technical factors such as data variance, the requirement for the application of advanced statistical and mathematical analyses, and difficulties associated with actually recognizing signature gene-expression patterns and discerning mechanisms. To aid the understanding of this powerful tool, its versatility, and how it is dramatically changing the molecular approach to biomedical and clinical research, this teaching review describes the technology and its applications, as well as the limitations and evolution of microarrays, in the field of organ transplantation. Finally, it calls upon the attention of the transplant community to integrate into multidisciplinary teams, to take advantage of this technology and its expanding applications in unraveling the complex injury circuits that currently limit transplant survival.

## Learning objectives

The purpose of this article was to review:

1. Microarrays, the basics
2. Evolution of microarray technology over time
3. The importance of microarrays in human biology
4. Microarray-based insights for the transplant physician
5. Current unmet biological questions in transplantation and how can we use microarrays to address them
6. Methods of applying microarrays to clinical practice
7. Limitations of using microarrays in clinical practice
8. The black-box of microarray data analysis

## Introduction

The completion of the human genome project has revolutionized translational medicine. High-throughput technologies, mapped to known target sequences, many with known functions, now permit investigators to interrogate the genome, transcriptome, proteome and metabolome systematically, and to assess genomic mutations, polymorphisms, epigenetic alterations, and micro-RNAs. These technologies herald the potential for us to translate their results into novel sensitive and specific diagnostic tests and less toxic therapeutics, with the anticipation of moving away from protocol-based approaches to personalized medicine. In this new era, investigators could potentially assess the molecular and pathophysiological characteristics of individual patients and their transplanted organs, tailor therapeutic regimens, and administer them based on these profiles. One of the key steps in this process will be the identification and validation of biomarkers. To date, microarray technologies are, perhaps, the most successful and mature methodology for high-throughput and large-scale genomic analyses.

## Microarrays, the basics

Microarray technology is based on the principle of complementary, single-stranded, nucleic acid sequences forming double-stranded hybrids; thus, in essence, it is a high-throughput Southern blot, where thousands of single-stranded sequences that are complementary to target sequences are synthesized, or spotted on to a small glass or membrane support. The gene probes on the array are either small (20–60 bp) single-stranded oligonucleotides, synthesized in situ (provided by Affymetrix, Agilent), or cloned complementary DNA (cDNA) amplified by polymerase chain reaction (PCR) and obtained by reverse transcription of messenger RNAs (mRNAs). Illumina uses designed oligonucleotide probes attached to beads that are deposited randomly on a substrate. The selection of microarray platforms will have important effects in the later stages, determining the complexity and flexibility of the data to be analyzed. *Care must be taken to choose a platform that will allow data to be analyzed and disseminated in the manners desired*. Experimental design and analysis are generally more straightforward with one-color microarrays.

**Fig. 1:**
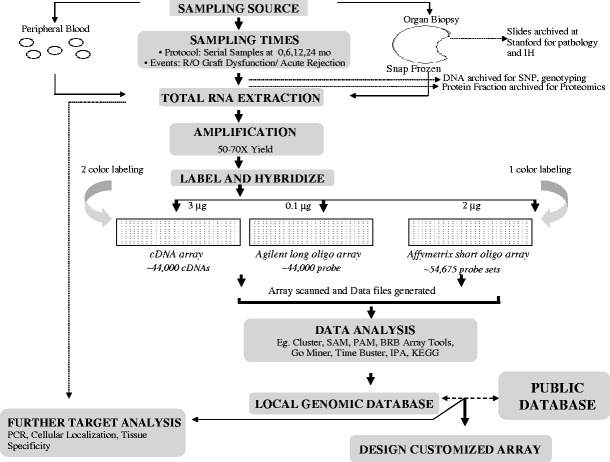
Schematic representation of DNA microarray technology. Total RNA is first isolated from the samples of interest; this test RNA and a reference RNA are then differentially labeled with fluorescent dyes and then competitively hybridized onto a printed DNA microarray. Images that are generated are then scanned, and the resulting fluorescence intensities are used for further data analysis. *IH* immunohistochemistry, *SNP* single nucleotide polymorphism, *SAM* significance analysis of microarrays, *PAM* prediction analysis of microarrays, *BRB* Biometric Research Branch, *GO* Gene ontology, *IPA* Ingenuity Pathway Analysis, *KEGG* Kyoto Encyclopedia of Gene and Genomes

In organ transplantation, a prospective study of the gene-expression profile of graft injury usually involves sample collection from tissue biopsy, blood and biofluids, such as urine, bile, or broncho-alveolar lavage, taken before, during, and after injury. A schematic presentation of microarray study is shown in Fig. 1. All collected samples are then subject to standardized protocols for RNA extraction [1]. Routinely, one assesses RNA quality control by looking for ribosomal RNA ratios of 260/280 > 2. The integrity of the RNA can also be assessed with Agilent 2100 Bioanalyzer, using RNA Nano Chips (Agilent Technologies), where the degradation of RNA can be determined by RNA integrity number (RIN) [2, 3]. RNA amplification techniques are often required for microarray analysis and are related to downstream genetic analyses when small sample input is used as starting material. Linear RNA amplification is a strategy that has been used successfully to generate adequate input RNA for molecular profiling studies. One method of linear amplification, termed amplified antisense RNA (aRNA) amplification [4], utilizes a T7 RNA polymerase-based amplification procedure that allows quantitation of relative gene expression levels from small tissue samples. With modification of the classic Eberwine method, Wang et al. [5] exploited a template switching effect at the 5′ end of the mRNA transcript to ensure the synthesis of full-length double-stranded cDNA. The most common aspects arising from the use of sample amplification, irrespective of whether the method confers linear or exponential amplification, include amplification efficiency, 3′ bias and length of aRNA/cDNA products, reproducibility, fidelity of maintaining relative transcript abundance, benefits of the use of amplified material versus non-amplified material, and disadvantages with amplification procedures [6].

RNA is next labeled with a detectable marker (fluorescent dye) and hybridized to an array containing individual gene-specific probes, in either a dual-color (sample and control pool with different colors, e.g. cDNA array) [7] or single-color (sample label only, e.g. oligonucleotide arrays) hybridization system [8]. The array is hybridized with the labeled sample(s) by incubation (usually overnight) and is then washed to remove non-specific hybrids. A laser excites the attached fluorescent dyes to produce light detected by a scanner, which generates a digital image from the excited microarray. The digital image is then processed by specialized software to transform the image of each spot into a numerical reading. This process finds the specific spot location and shape, summarizes spot intensities, and subtracts the surrounding background noise. To facilitate the comparison between the experiments and to compensate for differences in labeling, hybridizations and detection methods, a data normalization step is usually performed. This final numerical reading is proportional to the concentration of the target sequence in the sample to which the probe in the spot is directed. In competitive two-dye assays, the reading is transformed to a ratio equal to the relative abundance of the target sequence (labeled with one type of fluorochrome) from a sample respective to a reference sample (labeled with another type of fluorochrome). In the one-dye technologies, the fluorescence is commonly yellow, whereas, in two-dyes technologies, the colors used are green for reference and red for sample (although a replicate using dye-swap is often done for quality control). The appropriate choice of technology depends on experimental design, availability and cost.

## Evolution of microarray technology over time

Early microarray technology allowing the hybridization of high-density cDNA with samples on nylon membranes provided a technological step in the right direction, but this technology is limited in scope by the nature of the matrix supporting the clones. Two later innovations made possible the new microarray technologies. One was the use of solid supports, such as glass, which are much more amenable to miniaturization and fluorescence-based detection [7]. The second innovation was high-density oligonucleotides on glass wafers using photolithographic masking techniques. The latter approach is more versatile in its applications and has the key advantage that the oligonucleotides can be synthesized at will, allowing chips to be manufactured directly from sequence databases. Using this technology, Affymetrix’s short-oligonucleotide array (HG_U133plus2.0) has became one of the most popular platforms for research [9, 10]. Agilent has applied its ink printing technology to microarrays, which has enabled high-speed and quality production of oligonucleotide microarrays [11]. The Illumina BeadChip is a relatively new method with increasing usage. The essential element of BeadChip technology is the attachment of oligonucleotides to silica beads. The beads are then randomly deposited into wells on a substrate, such as a glass slide. The resultant array is decoded to determine which oligonucleotide-bead combination is in which well [12]. More recently, Affymetrix developed the GeneChip Exon Array, which offers a more complete and accurate picture of overall gene expression by enabling researchers to investigate the entire length of the gene, not just the 3′ end. This exon-level analysis on a whole-genome scale opens the door to the detection of specific alternative splicing events that may play a central role in disease mechanism and etiology. These arrays have greater than 99% coverage of sequences present in the RefSeq database, covering only well-annotated content [13]. The evolution of microarray technology is summarized in Table 1.

**Table 1:** The evolution of microarray technology (*n/a* not applicable)

Type of array	Number of probes or probe sets	Target spots	Manufacturer	Year invented
Nylon	5,000	cDNA	n/a	1996
Glass	40,000	cDNA	Stanford	1996
Glass	54,000	25mer oligonucleotides	Affymetrix	2000
Glass	46,000	60mer oligonucleotides	Agilent	2004
Exon Array	1 million exons	123mer oligonucleotides	Affymetrix	2005
BeadChip	50,000	79mer oligonucleotides	Illumina	2005

The overall differences in cDNA (e.g. Lymphochip) vs oligonucleotide-based arrays (e.g. Affymetrix, Agilent) [14] lie in the fact that the probe for cDNA arrays is 0.5–3 kb in length, and it is 15–70 bp in length for the oligonucleotide arrays. The oligonucleotide arrays can also perform genotyping studies and detect splice variants, in addition to mRNA profiling, but, unlike cDNA arrays, they require multiple probes per target, with greater spot consistency and less batch-to-batch variability.

## The importance of microarrays in human biology

Microarray technologies were initially designed to measure the transcriptional levels of RNA transcripts derived from thousands of genes within a genome in a single experiment. This technology has made it possible for one to relate physiological cell states to gene-expression patterns for studying tumors, disease progression, cellular response to stimuli, drug target identification and transplant injury mechanisms. For example, subsets of genes with increased and decreased activities (referred to as transcriptional profiles or gene-expression “signatures”) have been identified for acute lymphoblast leukemia [15], breast cancer [16], prostate cancer [17], lung cancer [18], colon cancer [19], multiple tumor types [20], organ transplantation [1], and drug response [21]. Moreover, because the pool of published data grows every day, integrated analysis of several studies, or “meta-analysis”, have been proposed in the literature [22]. These approaches detect generalities and particularities of gene expression in diseases.

More recent uses of DNA microarrays in biomedical research are not limited to gene-expression. DNA microarrays are being used to detect single nucleotide polymorphisms (SNPs) of the human genome (Hap Map project) [23], aberrations in methylation patterns [24], alterations in gene copy number [25], alternative RNA splicing [26], pathogen detection [27, 28] and micro-RNA [29].

Gene-expression profiles for prognostic classifiers are usually built by the correlation of gene-expression patterns, generated from specimens, with clinical outcome (e.g. acute rejection vs stable without rejection). Gene-expression predictive classifiers of response to treatment are generated by the correlation of gene-expression data, derived from samples taken before treatment, with clinical and pathological response to treatment. Although the identification of the most relevant information from microarray experiments is still under active research, well-established methods are available for a broad spectrum of experimental set-ups. The analysis of gene-expression data at the pathway and functional level, along with a systems biology approach, will provide deeper insights into the biological effects of complex disease states, such as in the organ transplant milieu, and will improve risk assessment of the same.

## Microarray-based insights for the transplant physician

It is challenging to dissect any allograft injury mechanism with single-gene studies because of the complexity of the mechanisms for renal allograft rejection with different immunosuppressive protocols and the spectrum of the response with immunological injury. Previously researchers have reported that expression of the cytotoxic molecules granzyme B and perforin has been associated with rejection and has been detected in blood [30], urine [31], and biopsy tissue samples [32, 33] in human and experimental studies. However, renal allografts transplanted into perforin or granzyme A or B “double knockout” (gene deletion) mice showed T cell-mediated rejection that was not mediated by perforin or granzymes [34], indicating the redundancy of the immune response during rejection.

The advent of microarray technology has enabled researchers to detect the expression of thousands of genes simultaneously, rather than measuring the expression of one gene at a time, and has unlocked information about disease heterogeneity that could not have been predicted by standard clinical or pathologic criteria. Pioneering studies of gene-expression profiles in breast cancer have identified the molecular classification of breast cancer into clinically relevant sub-types. This has provided new tools with which one can predict cancer recurrence and response to different treatments, and new insights into various oncogenic pathways and the process of tumor progression [35]. Subsequent microarray studies have changed the paradigm of approach in lymphoma [36], where the diffuse B-cell lymphoma was identified as having the worst prognostic outcome, and in kidney transplantation, where rejection sub-types with differential survival benefits and a prognostic role for focal B-cell infiltrates was identified for recalcitrant rejections [1]. Using cDNA microarrays, Hauser and co-workers [37] have determined the gene-expression patterns specific to living-donor vs deceased-donor kidneys and suggest that suppression of specific targets of inflammation in the deceased donor might be a promising intervention for abrogating post-ischemic acute renal failure.

These findings have brought a global paradigm shift from traditional hypothesis-driven experiments toward large-scale hypothesis generation and testing through clinical trials. In the past few years, there has been an increasing number of publications on solid organ transplantation, with particular emphasis on the heart and kidney. Supporting the results of earlier studies, microarrays have also corroborated the finding of known pathways in rejection injury, such as evidence of the dysregulation of the complement system [38, 39], interleukins [40], anti-human leukocyte antigen (HLA) allo-antibodies [41, 42] and solute transport genes [43], in allograft rejection.

**Fig. 2:**
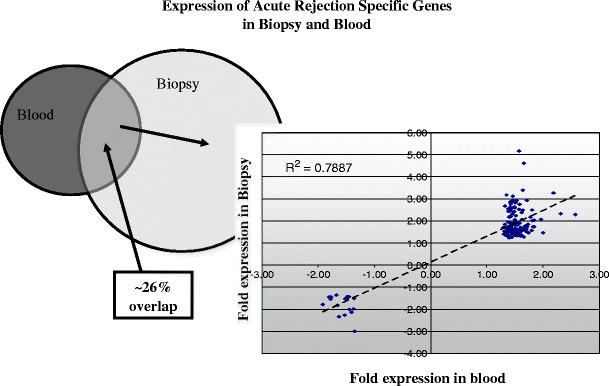
Correlation of acute rejection gene expression in biopsy vs blood. Significant genes for graft rejection are identified in blood and biopsy tissue (Sarwal et al., unpublished data) with low false discovery rates (q scores < 1% by significance analysis of microarrays (SAM) analysis (https://doi.org/www-stat.stanford.edu/∼tibs/SAM/)). The logarithmic fold expression values are shown on the X and Y axes. Only 26% of the significant genes overlap in the two tissue sources. These overlapping genes show much higher fold expression in tissue than in blood

The interrogation of minimally invasive or non-invasive biomarkers of graft injury has been more challenging than the direct interrogation of the transcriptional changes in the injured graft. Until recently, most gene-expression profile studies were performed on biopsy specimens. Transcriptional changes in peripheral blood during graft rejection demonstrate significantly disparate gene-expression changes from those of the inflamed graft, suggesting that the local response to inflammation and injury in the rejecting organ is highly localized. Additionally, the intensity (fold-change) and quantity (number of significant genes in rejection) of the rejection response in peripheral blood is much smaller than the corresponding response in the organ, even when biopsy and blood samples from the same patient are examined simultaneously [44] (Fig. 2). A recently published study [45, 46] using both microarray and reverse transcriptase-polymerase chain reaction (RT-PCR) to discriminate rejection from non-rejection in peripheral blood samples from heart transplant patients gave a reasonable correlation only with severe and high-grade tissue rejection. Further, this was only from samples taken later than 6 months after transplantation, even though the risk of rejection is highest during the first 6 months after transplantation [47]. When biopsy predictor sets were used on blood samples, these microarray data from blood did not give significant predictions [47]. Despite these limitations of peripheral blood sampling, efforts to examine this sample source for clinical monitoring continue to hold promise. The answer for increasing the sensitivity and specificity of biomarker detection in peripheral blood may lie in the more careful attention to improved methods of sample collection, storage, and processing [44].

Conventional wisdom holds that long-term allograft survival requires life-long immunosuppression. A tremendous advance in peripheral blood monitoring for immunosuppression customization may lie in the data emerging from studies on a highly selected group of organ transplant patients with spontaneous graft acceptance or prope tolerance in liver [48] and kidney [49]. Blood gene-expression profiles from transplant patient cohorts with tolerance, stable graft function and acute and chronic graft injury, as well as peripheral blood samples from healthy individuals, were analyzed on microarrays [49]. A tolerance-specific signature of 49 genes was identified in the kidney patients, which was strongly regulated by transforming growth factor-beta (TGF-β) signaling and cell cycle signaling. The tolerance signature in immunosuppression-free liver patients included genes encoding for gamma delta thymus (T)-cell and natural killer (NK) receptors, and for proteins involved in cell proliferation arrest. Importantly, 50% of kidney recipients on steroid monotherapy, and 8% of kidney recipients on triple-drug immunosuppression, also had the tolerance signature, suggesting that those patients may benefit from immunosuppression minimization [49]. We anticipate that, after further validation studies, these biomarkers might be useful as minimally invasive monitoring tools for guiding immunosuppression titration and might provide novel mechanistic insights into the acceptance mechanisms for renal and liver allografts. As there is only a single gene overlap between the renal and liver tolerance signatures, we can hypothesize that either there is sufficient redundancy in the system or there is some tissue (liver vs kidney) specificity. Key array-based published studies of transplantation are summarized in Table 2.

**Table 2:** Key array-based published studies in transplantation (*AR* acute rejection, *CAN* chronic allograft nephropathy, *TOL* operational tolerance, *MIS* minimum immunosuppression, *HTN* hypertension, *RVA* reno-vascular abnormalities, *EPO* erythropoietin, *LDN* laparoscopic donor nephrectomy, *DT* drug toxicity, *HTN* hypertension, *STA* patient with stable graft function)

Author	Journal	Array type	Tissue	Phenotype	Key findings
Human studies					
Brouard et al. [49]	Proc Natl Acad Sci U S A 2007	cDNA	Lymphocyte	TOL, AR, CAN, stable, MIS	AKR1C1, AREG, BRRN1, C1S, CCL20, CDC2, CDH2, CHEK1, DHRS2, DEPDC1, ELF3, HBB, IGFBP3, LTB4DH, MS4A1, MTHFD2, PARVG, PLXNB1, PODXL, PPAP2C, RAB30, RASGRP1, RBM9, RHOH, SLC29A, SMILE, SOX3, SPON1, TK1 and TLE4
Li et al. [44]	Physiol Genomics 2007	U133plus GeneChip	Blood	STA, AR	Globin genes onfounders in biomarker discovery from PAX gene samples for AR
Nagarajan et al. [89]	Clin Transplant 2007	cDNA	Peripheral blood	HTN, RVA, EPO	Hemoglobin zeta, G2, E1, CTGF, PLA2 G2A, PDGF-A, VEGF, CDH5, GDF1, TIE, TBRG1, EPS8, FIBP, EPOR, TFRC, STAT5, Jak2 and CLK1
Park et al. [90]	Transplantation 2007	U133A 2.0 GeneChip	Kidney biopsy	Fibrotic	STAT1, STAT2, proteasome subunit [beta]-type-8, Col1A1, FN1, phosphoinositide-3-kinase regulatory subunit-3, VCAM1, GRZMA, GBP1, IER3, HLA-DRbeta, IL-10, TGFB, IFNG, IL-6 and FoxP3
Mas et al. [91]	Transplantation 2007	U133A 2.0 GeneChip	Kidney biopsy, peripheral blood, urine	CAN	TGF-beta, laminin, gamma 2, metalloproteinases-9, collagen type IX alpha 3, immunoglobulins, cytokine, chemokines receptors, EGFR, FGFR2, AGT, EGFR and TGFB
Morgun et al. [47]	Circ Res 2006	Oligonucleotide	Heart biopsy, kidney and lung	AR, infection	CCL18, TRB, LTB, ITGB2, HA-1, CORO1A, IGKC, RARRES3, CCL5, HLADRB3, STAT1, C1QA, GMFG, CD74, CD14, PSCD4, BTN3A3, HLA-F and UBE2L6
Hotchkiss et al. [55]	Transplantation 2006	U133A GeneChip	Kidney biopsy	CAN	TGF-B, thrombospondin 1, PDGF, integrins, MMP7, C4B, properdin, VCAM1, Annexins, VEGF, EGF and FGF
Kurian et al. [54]	Transplantation 2005	U133A GeneChip	Kidney biopsy	LDN	HIF1a, HIF1B, TNF, TNFR, TGF-B, FGF, integrins, MMP, elastin, GHRH and VEGF
Eikmans et al. [53]	J Am Soc Nephrol 2005	HG U95Av2 GeneChip	Kidney cortex	CAN	Surfactant protein-C (SP-C), S100 calcium-binding protein A8 (S100A8), S100A9 and immuno-globulin genes
Melk et al. [62]	Kidney Int 2005	cDNA	Kidney cortex	Renal aging	NADH dehydrogenase, APO, kynureninase PAH, dynein, CLDN8, MMP7, fibulin, tenascin, CSPG2, SERPINA3, immunoglobulins, somatostatin receptor, THY1, natriuretic peptide receptor and SLC solute transporter family
Zhang et al. [92]	Clin Transplant 2004	HG U95Av2 GeneChip	Lymphocyte	Stable transplant	Membrane-type matrix metalloproteinase 1, SH3 binding protein, MEA6, TOB family 4, RBP2, IL-1A, Argininosuccinate synthetase, Brain and nasopharyngeal carcinoma, NSG-x, hVH-5 and Eosinophil Charcot-Leyden crystal protein
Mansfield et al. [14]	Am J Transplant 2004	cDNA	Kidney biopsy	AR sub-types	MIP-1, CCR5, CX3CR1, DARC, SCYB10, SCYA5,SCYA3, SCYA13, SCYA2, IL2RB, IL6R, IL16, 1L15R, DEFA1, DEFB1, SCYA2, SCYA5, MST1, STAT1, STAT6, CD69, MAL, NFATC3, Annexins, CASP10, PECAM1 and VCAM1
Hauser et al. [37]	Lab Invest 2004	cDNA	Kidney biopsy	Donor source	Complements, LTF, NK4, VCAM1, interleukins, HLA, BCL6, GPX2,FBP1, PCK2, SORD, APOA4, CYP3A7, FABP1, APOM, CYP3A4, HIF1A, STAT1,TIMP1, ADAMTS1, TNFSF10 and CDC25B
Kainz et al. [93]	Am J Transplant 2004	cDNA	Kidney biopsy	Donor source	Osteopontin, SOD2, RARRES1, chemokine ligand 1, antileukoproteinase, STAT1, CDH6, SPP1, SERPINA3 and GPX2
Flechner et al. [56]	Am J Transplant 2004 (a)	Oligonucleotide	Kidney biopsy	CAN, drug effect	TGFB, TNFA, PDGF, ICAM, VCAM1, integrin B, MCP-1, CCR2, MPI-3B, MHC, MMP, TIMP1, RANTES, VEGF, collagen III, Angiotensin II receptor, TSP and FN1
Flechner et al. [94]	Am J Transplant 2004 (b)	HG U95Av2 GeneChip	Kidney biopsy, peripheral blood	AR	AIF, CD14, CD163, CD2, CD3D, CD48, CD53, chemokines, interleukins, C1q, immunoglobulins, INFG, TCR TNF, and HLA
Donauer et al. [57]	Transplantation 2003	cDNA array		CAN	AQP2, AQP3, lipoprotein lipase, PML-2, Napsin 1, precursor, Flotillin-1, Type IV collagenase, Hepatocyte growth factor activator inhibitor, RIG-like 7–1, MECI-1, PGER, TEM8, MHC class I, C1s and immunoglobulins
Higgins et al. [76]	Mol Biol Cell 2004	cDNA	Cortex, medulla, papillary tips,	Normal	Identify patterns of gene expression in discrete portions of the normal kidney
Sarwal et al. [1]	N Engl J Med 2003	cDNA	Kidney biopsy, pediatrics	AR, CAN, DT and infection	TCR, HLA class II, HLA class I, immunoglobulins, lactotransferrin, chemokines, CD20, CD34, IGF1R, TNFR, MST1, NK4, duffy antigen/chemokine, receptor, STAT1, TGFR1, granzyme A, perforin, IL2R, CD53, lymphotoxin, lymphotoxin R, NFKB1, CD59, IFNGR1 and annexins
Scherer et al. [58]	Transplantation 2003	HG U95Av2 GeneChip	Kidney biopsy	CAN	Keratin tumor suppressor candidate 7, OS9(APRIL), G-protein gamma7, protein/cell adhesion molecule-like, GRB2-associated binding protein 1, and PRLR
Chua et al. [95]	Am J Transplant 2003	cDNA	Kidney biopsy	AR/anemia	Hb-zeta, Hb-beta, Hb-alpha2, FOLR2, FOLR3, CAH1, immunoglobulins, GPX1, and lactotransferrin
Zhang et al. [96]	Transplant Proc 2002	Oligonucleotide	Lymphocyte	Stable transplant	CD80, interleukins, CD44, CD40L, CD40, VLA-5, LFA-1, TCR alpha, Lck, calcineurin, PKC, IFNG, LFA-1, TCR alpha, Lck, calcineurin, PKC, IFNG, TGFB, TNF-alpha, TNFR1, G-CSFR and PDGF receptor,
Akalin et al. [97]	Transplantation 2001	Hu6800 GeneChip	Kidney biopsy	AR	HuMig, TCR RING4, ISGF-3, CD18
Animal studies					
Kusaka et al. [98]	Transplantation 2007	Agilent rat oligonucleotide array G4130A	Kidney allografts, T lymphocytes	Brain death donor	Gro1, IP-10, p53, NF kappa B, Myc, Jun, c-fos, LCN2 and SPP1
Berthier et al. [99]	Kidney Int 2006	230 A GeneChip	Kidney allografts	CAN	MMP-11,-12,-14, ADAM-17, TIMP-1,-2 TGF-B, MMP-9, meprin and MMP-24
Djamali et al. [100]	Transplantation 2005	mouse stress toxicity GEArray	Kidney allografts	CAN	ANXA5, CASP1, CASP8, TNFRII, TRAIL, FASL, BAX, inducible nitric oxide synthase, cytochrome p450 4A, [alpha]-crystalline B, heme-oxygenase II, SOD, HSP60, HSP27, BCL-X and metallothionein
Schuurs et al. [101]	Am J Transplant 2004	Oligonucleotide	Kidney allografts	HTN brain death	Water channel AQP-2, selectins, IL-6, oc-B-fibrinogen, KIM-1, HO-1, Hsp70, MnSOD2, ATF-3, EGR-1 and PIK3R1
Einecke et al. [43]	Am J Transplant 2007	Oligonucleotide	Mouse kidney	Rejection	SLC2a2, SLC1a1
Leonard et al. [102]	FASEB J 2006	HG U95Av2 GeneChip, murine U77A	Mouse kidney, human proximal tubular epithelial cells	Ischemia reperfusion injury	In mouse model: ALDH1A1, ALDH1A7, GSTM5, GSTA2, GSTP1, NQO1 and Nrf2. In human: Nrf2 is up-regulated on reoxygenation
Famulski et al. [60]	Am J Transplant 2006	Oligonucleotide	Mouse kidney	AR	Define IFNG-dependent, rejection-induced transcripts (GRITs) in mouse kidney allografts. IFNG inducible: CXCl9, UBD and MHC
Einecke et al. [43]	Am J Transplant 2007	Oligonucleotide	Kidney allografts, T lymphocytes	Rejection	Cytotoxic T lymphocyte-associated transcripts (CATs): CD2, CD3g, GZMB, TCRB, MES

## Current unmet biological questions in transplantation: how can we address them?

Whilst great advances have been made in the discovery of putative biomarkers in transplantation, disappointingly few have been translated into clinically applicable assays; much of this is due to a lack of well-designed clinical validation studies. The most important challenge is having well-designed validation and varying endpoint definitions. To adapt molecular endpoints from single-gene studies as representative of a particular mechanism of toxicity/injury often assumes that a postulated mechanism must be known beforehand, and this may result in “over-fitting” of the data, making the inference not entirely accurate.

### Chronic allograft nephropathy what are the early injury pathways?

Chronic graft injury, potentially an indolent immune response resulting in slow deterioration of organ function, characterized pathologically in the kidney by tubular atrophy, interstitial fibrosis, and fibrous intimal thickening of the arteries, has a relatively transcriptionally homogeneous response of tissue fibrosis, when investigated at the time of the established injury [1]. Although there is a general consensus on the patient criteria for chronic allograft nephropathy (CAN), it is not universal. Despite the many presumed triggers for this injury (alloimmune responses, donor age and tissue quality; brain death; preservation/reperfusion injury; post-transplantation and systemic stresses in the recipient environment) [50], early injury triggers that could provide drug targets for manipulation of injury progression have not been identified in cross-sectional human studies. Animal models, where injury mechanisms can be segregated better, have been useful to study [51], but careful design of clinical trials to ascertain longitudinal and evolutionary studies on graft injury may be required. Given the difficulty of recipient consent for multiple post-transplantation biopsies, the discovery of biomarkers specific to CAN has been challenging. Most of the published reports (Table 2) are from a single sample time point [52–55]. However, with careful study design, controlled studies have been performed using sequential (paired) patient samples [56–58]. These studies are limited by relatively small sample size, non-standardized protocol biopsies, and few sample collection time points; perhaps the field will be led by organs where performance of these protocol biopsies is almost standard of care, e.g. heart and lung transplantation. Additional issues that would be important in the design of clinical validation studies for biomarkers would be: a prospective nature of sample identification, also allowing for samples that could be then used for prediction of the clinical events, maintaining homogeneity of patient sub-groups and disease pathology, similar immunosuppression regimes and controlling for patient demographics and the use of other concomitant treatments.

The limitations of microarray studies and the importance of well-designed validation strategies have been demonstrated by microarray applications in cancer. In an attempt to predict prognoses of cancer patients on the basis of previously published DNA microarray studies, re-analysis of data from the seven largest published studies showed that the list of genes identified as predictors of prognosis was highly unstable; the selection of training sets strongly affected molecular signatures [59], and biomarkers from the training sets did not perform as well in independent validation studies. The most important challenge in translational transplantation research is the lack of a true gold standard for the classification of disease in organ transplantation. The current histologic classification of deteriorating organ transplants has many limitations, including arbitrary cut-off points and poor inter- and intra-observer reproducibility. This makes identification of informative samples sets very difficult, resulting in the difficult generation of microarray-based hypotheses in transplantation.

### Acute rejection prediction and immunosuppression customization

Acute rejection (AR) depends on an orchestrated immune response to histocompatibility antigens expressed by the grafted tissue. The redundancy of AR mechanisms and the problems with peripheral blood transcriptional analysis (outlined above) has made it difficult for reliable biomarkers to be developed for prediction of allograft rejection and its outcome, irrespective of immunosuppression usage, concomitant infection, recipient age or organ type. The delineation of AR from antibody-mediated rejection (AHR, also termed humoral rejection) is still debated. Though effector mechanisms primarily responsible for the rejection process classically involve activation of effector T cells and memory T cells, alternative mechanisms of acute rejection also recruit (to varying degrees) B-cells, natural killer cells, eosinophils and neutrophils, antibody-mediated rejection is currently thought to play a role in approximately 33% of AR episodes [42]. Thus, though numerous markers of different biologic pathways have been evaluated as diagnostic and prognostic tools to serve this purpose in human [47, 56] and animal organ transplantation [60, 61], no biomarkers have become firmly established for prediction of acute rejection. It is unlikely that a single biomarker will meet all clinical needs, such as non-invasive diagnosis and prediction of transplant rejection and survival, given the clinical confounders in the recipients’ post-transplantation environment. Microarrays may, over time, offer multiple markers as gene-based tests, and combining these with the genes found for graft tolerance [49] may enhance future patient monitoring and enable individualized risk-adapted patient care.

### Limited donor source—how can we expand this?

Finding ways to use kidneys more effectively for transplantation has the potential to extend the donor pool using dead donors who meet the Standard Criteria for Donation (SCD) as well as those from Expanded Criteria Donors (ECDs), created by the United Network for Organ Sharing in 2002. Higher-risk donor organs, once considered unsuitable, could also be transplanted safely. With regard to higher-risk donor organs, the question remains: Are all kidneys from older donors equivalent with regards to biological and cellular health? An array-based analysis of kidneys from a wide spectrum of ages (8 months to 80 years) [62] suggested that, though older kidneys appeared to have increased extracellular matrix turnover and a non-specific inflammatory response, combined with a reduction in processes dependent on energy metabolism and mitochondrial function, these results did not always correlate with chronological age. Extension of expression data into hypothesis testing for correlation studies of older kidneys with good vs poor “transcriptional health” with post-transplantation function, may offer a means to potentially expand the donor pool. To date, these studies have not been performed.

## Methods of applying microarrays to clinical practice

### Identification of differentially expressed genes between sample groups

Identifying whether genes have increased or decreased in expression between two or more groups of samples is the most common and basic type of analysis and provides a simple characterization of the specific molecular differences that are associated with a specific biological phenotype. Using power analysis, it is possible to estimate the number of samples required to identify a high percentage of truly differentially regulated genes between sample groups. Unsupervised analysis [63] is a useful means to assess, a priori, the inter- and intra-group differences or similarities. Unsupervised analysis is based on the assumption that co-expressed genes have the potential to be regulated by same transcriptional factors or to have similar biological functions. Examples of unsupervised analysis methods are hierarchical clustering [64], R (https://doi.org/cran.r-project.org), the Gene Expression Profile Analysis Suite (GEPAS) [65], The Institute for Genomic Research (TIGR) T4 [66, 67], GeneSpring [68] and Genesis [69]. Typically, genes are represented on the y-axis, whereas samples are drawn on the x-axis, and a dendrogram on each axis shows the degree of relatedness of samples and genes to each other. A color-coded matrix (heat map), where samples and genes are sorted according to the results of the clustering, is used to represent the expression values for each gene in each sample and is the basis of many of the published microarray figures. Though *fold-change* in fluorescence intensity (expressed as the logarithm (base 2 or log_2_) of the sample divided by the reference), is often used descriptively, it fails to ascertain the true significance of small but significant changes in gene-expression levels. Univariate analysis measures of significance are preferred, e.g. for data sets with normalized distribution, the *t*-test and analysis of variance (ANOVA) test can be used, and for data without normalized distribution, the Wilcoxon or Mann–Whitney tests are often used. Another commonly used measure of significance testing is significance analysis of microarrays (SAM) (https://doi.org/www-stat.stanford.edu/~tibs/SAM/). It should be pointed out that the methods given in this review are a selection from many others possible.

**Fig. 3:**
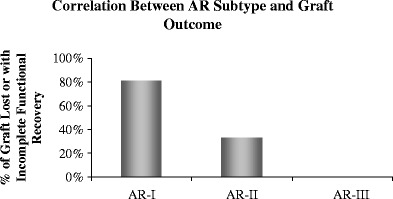
Correlation between AR sub-type and graft outcome. Analysis of the recovery of graft function over time revealed that grafts with AR that were clustered in the AR-I transcriptional sub-group had significantly poorer functional recovery than those classified as either AR-II or AR-III [1] (*P* = 0.02). *P* values were calculated from Kaplan–Meier survival analysis. Data are for grafts with incomplete functional recovery in the analyses according to sub-type of AR, where 80% of AR-1 and ∼40% of AR-II had incomplete recovery of serum creatinine to baseline values 6 weeks after treatment of the rejection episode. All AR-III episodes recovered graft function by the same definition

- *Determining biomarkers for clinical phenotypes of disease*
  The identification of gene-expression “signatures” associated with diseases categories is called biomarker detection or supervised classification. As the biomarker panel needs to be predictive of disease class or clinical outcome, learning and validation sets of samples are required, making the sample size relatively large for this type of analysis. Prediction analysis of microarrays (PAM, https://doi.org/www-stat.stanford.edu/~tibs/PAM/) is a powerful tool that can be adapted for this use. The selection of a unique list of genes by this approach does not, in and of itself, offer sufficient knowledge for one to understand the biology of a given system, suggesting the necessity to incorporate biological knowledge into array analysis. Recent approaches to microarray analysis address the limitations of conventional bioinformatics approaches by enriching the analysis with knowledge of biological processes [70]. This approach has the advantage over classical bioinformatics approaches that the feature selection step can be performed based on data that are completely independent of the clinical samples used for the analysis. This strategy is very promising, especially in disease states that are not easily classified into clear distinct categories, as is the case in clinical transplantation. Some ways to do this are either to use commercially available software (Ingenuity Pathway Analysis: https://doi.org/www.ingenuity.com/; Pathway Studio: https://doi.org/www.ariadnegenomics.com/) or to use hypergeometric enrichment analysis from published data sets of biologically relevant experiments [1, 49].
- *Microarray data for survival analysis*
  Biomarkers that correlate with survival times are a very important objective in the analysis of microarray data. Selected genes can be combined with clinical classes and incorporated into regression models to detect variations in survival times using both the Kaplan–Meier method and statistical tests. An example of this is shown where the gene-expression microarray data for different molecular rejection groups (AR-1, AR-2, and AR-3) [1] segregate by the performance for recovery of graft function 4–6 weeks after treatment intensification for the rejection episode (Fig. 3). Different linear regression models can be tested with independent variables (time, drug levels, and graft function) and dependent variables (genes) to ascertain any association between gene-expression and clinical variables.
- *Other applications of microarrays in clinical practice*
  Commercially available microarrays can detect single nucleotide polymorphisms (SNPs), which are an important tool for identifying genetic loci linked to complex disorders [71]. Unfortunately, the number of SNPs covered by the array-based methods is fewer than 1% of the known SNPs deposited in the public databases. *Altered methylation patterns* in genomic DNA can also be identified by microarrays, by the use of methylation-sensitive restriction enzymes to generate fragments enriched with either unmethylated or methylated CpG sites. Epigenetic phenomena, such as cytosine methylation, histone acetylation and phosphorylation, control the activation and deactivation of genes, such that genes methylated in their promoters can become inactive and can predispose individuals to cancers [67]. *Chromatin immune-precipitation (ChIP-on-chip)* assays [72, 73] can allow the estimation of alterations in the expression of transcription factors in several diseases (e.g. c-Myc is known to be differentially expressed in a variety of cancers [74]). *Pathogen specific microarrays* have been generated [27, 28] and can allow the direct interrogation of specific pathogens on an array-based platform.

## What limits the use of microarrays in clinical practice?

Gene-expression profiling studies of renal transplantation are highly complex, and the successful execution of such studies requires close collaboration between physicians, molecular biologists and bioinformatictists. The most important challenge is the lack of a true gold standard for the classification of disease in organ transplantation. The current histologic classification of deteriorating organ transplants has many limitations, including arbitrary cut-off points and poor inter- and intra-observer reproducibility. The classical approach to microarray analysis, which starts with the identification of genes that differentiate between two sample groups, depends on the assumption that distinct disease entities exist and that we know, with certainty, what these classes are. While the cancer literature may be able to rely on disease classification based on outcome data, the situation in organ transplantation is much more complicated, with many overlapping disease processes occurring simultaneously. If microarray analysis in clinical transplantation starts with a classification based on a flawed clinical gold standard, the results of the microarray study will not be any better than histologic examination and may even be misleading. Currently, there is no simple solution to this problem.

- *Quality control*
  The high-throughput nature of this technology, combined with the expected large numbers of data, result in a high risk for error. With the increasing use of genomic studies in transplantation, there is a need to control for various confounder effects that obscure biomarker discovery in graft rejection. In view of many concerns raised, the US Food and Drug Administration (FDA) lunched the Microarray Quality Control (MAQC) project. An excellent correlation of gene expression of human reference RNA (Stratagene) and human brain reference RNA (Ambion), across seven different array platforms, across five different laboratories, using three different amplification protocols [75], was shown in this study. Thus, while the need for quality control is a limitation for array studies, recognition of means to address this could turn this around as a benefit, resulting in the generation of robust datasets that could be queried with confidence by multiple users.
- *High cost*
  At present, because of the sophistication of microarrays, this is a costly technology available only in selected laboratories. Microarray technologies, however, are rapidly improving, and the costs of the technique continue to fall, thus paving the way for wider access and more generalized usage.
- *Sampling variability*
  Particularly for renal transplant biopsies, differing amounts of cortex vs medulla represented in a sample can affect the pattern of gene expression of a sample. Therefore, one can cross-reference a publicly available gene list specific for different compartments of kidney to minimize false clustering of samples [76]. There is also the problem of variable sample pathology. If only one biopsy core is being used for microarray analysis, it will be necessary to identify transcriptional changes that are more global and robust than patchy cellular interstitial infiltration, such as effects of cytokines on the renal tissue or global interstitial changes. mRNA is a very fragile molecule that can be degraded within minutes of surgical procedure [77], drastically affecting the interpretation of microarray data [78]. Moreover, subtle variations in biopsy handling and method of RNA extraction from samples can result in different levels of gene expression [78].
- *Difficulty in detecting some disease processes in transplantation by microarrays*
  Existing collagen, readily visible to the pathologist, is not necessarily associated with mRNA changes if the process of active fibrogenesis is complete. Small cell populations that make a major contribution to disease might give only a weak signal in transcriptome studies of whole biopsies or unseparated blood. Antibodies produced in lymphoid tissues could damage the kidney without any mRNA being detectable in the kidney. Microarray analysis cannot offer insights into these critical cellular and molecular processes in the tissues.
- *Discrepancy in array studies*
  Weak overlap exists between gene lists from individual studies of similar phenotypes in transplantation. The disparity among microarray data can be attributed to several factors: differences in microarray platforms with differing gene sets; weak statistical power and small sample sizes; biological variance because of variability in patient characteristics; experimental variance including lack of uniform protocols for study design, sample collection, RNA processing, and sample labeling and hybridization; different tools for data processing and statistical analysis (Table 3); variable thresholds for data filtering; varying stringencies for false discovery rates and statistical significance; and different data analysis methods. Nevertheless, a recent study compared microarray data for rejection across platforms, samples, and laboratories with some success. A gene set for acute rejection prediction generated from a heart biopsy [47] was used to predict previous published data for kidney biopsy [1, 56] and lung broncho-alveolar lavage cells [79].
- *Confounders exist in microarray experiments*
  Previous studies have demonstrated that the abundance of globin genes in whole blood may mask the underlying biological differences in whole-blood samples. In a comparison of gene-expression profiles of peripheral blood, using different protocols of sample preparation, amplification and hybridization on the Affymetrix platform, we demonstrated that the globin reduction method is not sufficient to unmask clinically relevant, rejection-specific, transcriptome profiles in whole blood. Additional mathematical application for globin gene depletion improves the efficacy of globin reduction but cannot remove the confounding influence of globin gene hybridization [44]. Other problems of analysis of blood may be more serious than the globin issue: the massive changes in cell populations caused by illness, surgery or infection make it difficult to define small changes in specific mRNA levels. It will be challenging to distinguish the blood signal for the alloimmune response from such common non-specific changes.

**Table 3:** List of pitfalls in microarray analyses and solutions (*SVD* singular value decomposition, *Cy* cyanine, *qPCR* quantitative polymerase chain reaction)

Pitfalls in microarray analysis	Solutions
Data variability, particularly for genes with low expression levels	Use replicate arrays to reduce false positives
Small sample amounts which limit replication	Use of amplified RNA (aRNA)
Expression bias due to amplification	Use improved protocols with single-roundamplification
Difficult to control input RNA amounts accurately	Use of normalization standard and two-color labelingstrategy to minimize
Spot quality may vary	Use stringent data-filtering criteria to assess signal/noise ratio and spot signal consistency
Lot-to-lot variation in PCR yield on cDNA arrays	Use data-filtering methods such as SVD to reduce batch biases (see text)
Hybridization efficiency varies with different probes	Use long-oligonucleotide arrays to minimize selected hybridization artifacts
Unequal labeling efficiency of Cy3 and Cy5 dyes	Use reciprocal labeling to confirm observations or use single-dye labeling system
Small numbers of samples and very large numbers of genes analyzed may contribute to false discovery	Confirm mRNA measurements using independent test methods such as qPCR and independent samples
Heterogeneity within study groups may contribute to false discovery	Use statistical modeling such as logistic regression to combine multiple genes
Protein expression levels and function not measured	Conform with protein expression methods (e.g. immunohistochemistry, protein arrays)

## The black-box of microarray data analysis

All published microarray studies should be made publicly available through the internet, on proprietary websites and in public microarray database repositories, and should generally follow the minimum information about a microarray experiment (MIAME) compliance format [80] or microarray gene-expression markup language (MAGE-ML) [81].

Many freely available software tools are now available for microarray data analysis. Though not completely intuitive, they have extensive manuals that can take a relatively inexperienced user towards the rapid understanding of their application for data analysis, ranging from image analysis, visualizations, differential expression, principal component analysis, clustering, classification, regression and survival analysis. Examples of some of these selected analytical methods are:

- The Gene Expression Profile Analysis Suite (GEPAS; https://doi.org/www.gepas.org)
- The Institute for Genomic Research (TIGR; https://doi.org/www.tigr.org/software/microarray.shtml)
- Significance analysis of microarrays (SAM; https://doi.org/www-stat.stanford.edu/tibs/SAM/),
- Prediction analysis of microarrays (PAM; https://doi.org/www-stat.stanford.edu/tibs/PAM/),
- Expression Profiler: next generation (EP:NG; https://doi.org/www.ebi.ac.uk/expressionprofiler),
- Bioconductor (https://doi.org/www.bioconductor.org),
- Cancer gene expression data analyzer (caGEDA; https://doi.org/bioinformatics.upmc.edu/GE2/GEDA.html),
- Analysis of microarray data (AMIADA; https://doi.org/dambe.bio.uottawa.ca/amiada.asp),
- GenePattern (https://doi.org/www.broad.mit.edu/cancer/software/genepattern/),
- Genesis (https://doi.org/genome.tugraz.at/Software/Genesis/Description.html)

A comprehensive discussion of different analytical strategies for microarray analyses are beyond the scope of this review.

The annotation of probes on microarrays is problematic for data analysis, which is shown in a particular commercial microarray design in which the number of probes associated with a given gene changes over time. These changes concern approximately 5% of the probe sets across the history of annotation releases over a 2-year span [82]. For Affymetrix Mouse 430 A/B, 13,699 out of 45,000 probes changed gene names from 2003 to 2004, and 2,277 (5%) probes changed annotation by their Entrez Gene identifiers [82]. Similarly, in human array platforms, e.g. for the HG-U133plus Affymetrix chip, unreliable representative public identifiers were seen for 18.2% [83] of the probes.

Probe redundancy is an additional problem (each transcript is probed by multiple oligonucleotide probes). This could potentially be caused by an annotation problem, with at least 5% of misannotation in each generation of the platform [82], e.g. multiple probes may assign to multiple genes or a single probe may map to multiple genes or Entrez IDs. The attention of manufacturers should be drawn to the maintenance of annotation accuracy and to the reduction of the number of probes required for each gene, attempting to choose the most representative probe/s. In addition, different portions of probe sets contained unreliable representative public gene IDs, with multiple genome hits. Harbig et al. [84] recently reassigned the probe sets on the Affymetrix platform, on the basis of each 25-mer probe sequence, and found that a large percentage of probe sets did not actually bind fully to a gene. They concluded that the set of probes assigned to be an official probe set is a problem with the Affymetrix platform. This is also a significant problem that may also be an issue with the other platforms, i.e. the sequence of a gene changes as additional information becomes available.

There are also different levels of detection (background over noise) for probes or probe sets, with specific criteria for each platform, which can have an impact on downstream analyses. Some of the different criteria used for a probe detection for different array platforms are discussed: *Agilent* cut off, absolute value of log_2_ red channel/green channel > 0.5 for at least one array; *cDNA* mean of channel 1 intensity/media background intensity > 1.5, and/or normalized mean of channel 2 intensity/media background intensity > 1.5; *Affymetrix* using a perfect-match-only model, the value for each probe or probe sets are extracted after background subtraction.

The exiting tools for converting the probe ID between microarray platforms are very limited. The difficulty in reusing data lies with the mapping of probes to established gene identifiers. Therefore, microarray results need to be re-evaluated periodically with the latest probe annotations. Most recently, a tool (Array Information Library Universal Navigator, AILUN, https://doi.org/ailun.stanford.edu/) was developed by a Stanford University group that re-annotates all gene-expressions/proteomics data from the Gene Expression Omnibus (GEO; https://doi.org/www.ncbi.nlm.nih.gov/geo/), which is a public repository for gene-expression and other high-throughput experimental data covering numerous platforms and species. The AILUN server builds a universal identifier ID table by relating all probe IDs to Entrez Gene IDs on a monthly basis, and it is the first tool available that allows researchers to compare microarray data across different platforms and map genes across species [85]. It also provides an opportunity for further discovery of complicated disease processes using more samples that have been deposited in GEO. The choice of processing method has a major impact on differential expression analysis of microarray data [86]. Some statistical issues should be given consideration in data analysis, such as class comparison, class prediction and class discovery [87]. When microarray data are being compared, various factors influence the agreement between studies, such as different technologies and platforms, statistical analysis criteria, protocols, and laboratory variability [88].

## Conclusions

High-throughput DNA microarray technology has been increasingly applied in kidney transplantation to classify molecular sub-types, to predict outcome and the response to treatment, and to identify novel therapeutic targets. Although results hold promise, this technology will not have a full impact on routine clinical practice until there is further standardization of techniques and optimal clinical trial designs to set up higher volume validation studies for the generated biomarkers. Owing to substantial disease heterogeneity and the number of genes being analyzed, collaborative, multi-institutional studies are required to accrue enough patients for sufficient statistical power. Customized arrays or multiplex PCR for informative biomarkers can then be applied to the clinics for event prediction, treatment stratification, immunosuppression customization and improved graft and patient survival.

Our scientific environment is ripe for research-based implementations of integrative tools that support knowledge-based data mining. This integration can provide the cornerstone of research in the coming years. Developments in this area will require close interdisciplinary collaboration and will lead not only to the integration of data and knowledge, but also to computer-supported experiments and to knowledge generation platforms, thereby closing the loop of data gathering, hypothesis generation and hypothesis testing.

## Questions

(Each question can be either true (T) or false (F). Answers appear after the reference list)

1. Microarrays: the basics
  - Microarrays are gridded probes on glass slides
  - Microarrays consist of complementary DNA or synthesized oligonucleotides
  - cDNA arrays consist of short probes 20–60 bp in length
  - Oligonucleotide arrays use a common reference and use two-color hybridization schema
2. The importance of microarrays in human biology lies in our ability to use them for
  - doing polymerase chain reactions
  - genotyping by checking for single nucleotide polymorphisms
  - searching for changes in the copy number of certain genes, specifically in cancers
  - assessing peptide mass
3. Microarray studies in transplantation have revealed to date that
  - there is molecular heterogeneity in acute graft rejection
  - there are many differences in the etiologies of chronic graft injury
  - B-cells can play a critical role in the rejection process and outcome
  - biomarkers can be used potentially for immunosuppression minimization
4. Limitations of using microarray in clinical practice lie in the following areas
  - ease of analysis
  - lack of hybridization specificity of cDNA arrays
  - problems with annotation of gene names on array platforms
  - no need to amplify human samples

## References

[1] Sarwal M, Chua MS, Kambham N, Hsieh SC, Satterwhite T, Masek M, Salvatierra O (2003). Molecular heterogeneity in acute renal allograft rejection identified by DNA microarray profiling. N Engl J Med.

[2] Schroeder A, Mueller O, Stocker S, Salowsky R, Leiber M, Gassmann M, Lightfoot S, Menzel W, Granzow M, Ragg T (2006). The RIN: an RNA integrity number for assigning integrity values to RNA measurements. BMC Mol Biol.

[3] Imbeaud S, Graudens E, Boulanger V, Barlet X, Zaborski P, Eveno E, Mueller O, Schroeder A, Auffray C (2005). Towards standardization of RNA quality assessment using user-independent classifiers of microcapillary electrophoresis traces. Nucleic Acids Res.

[4] Van Gelder RN, von Zastrow ME, Yool A, Dement WC, Barchas JD, Eberwine JH (1990). Amplified RNA synthesized from limited quantities of heterogeneous cDNA. Proc Natl Acad Sci USA.

[5] Wang E, Miller LD, Ohnmacht GA, Liu ET, Marincola FM (2000). High-fidelity mRNA amplification for gene profiling. Nat Biotechnol.

[6] Nygaard V, Hovig E (2006). Options available for profiling small samples: a review of sample amplification technology when combined with microarray profiling. Nucleic Acids Res.

[7] Schena M, Shalon D, Davis RW, Brown PO (1995). Quantitative monitoring of gene expression patterns with a complementary DNA microarray. Science.

[8] Eberwine J, Yeh H, Miyashiro K, Cao Y, Nair S, Finnell R, Zettel M, Coleman P (1992). Analysis of gene expression in single live neurons. Proc Natl Acad Sci USA.

[9] Fodor SP, Read JL, Pirrung MC, Stryer L, Lu AT, Solas D (1991). Light-directed, spatially addressable parallel chemical synthesis. Science.

[10] Chee M, Yang R, Hubbell E, Berno A, Huang XC, Stern D, Winkler J, Lockhart DJ, Morris MS, Fodor SP (1996). Accessing genetic information with high-density DNA arrays. Science.

[11] Hsieh HB, Fitch J, White D, Torres F, Roy J, Matusiak R, Krivacic B, Kowalski B, Bruce R, Elrod S (2004). Ultra-high-throughput microarray generation and liquid dispensing using multiple disposable piezoelectric ejectors. J Biomol Screen.

[12] Gunderson KL, Kruglyak S, Graige MS, Garcia F, Kermani BG, Zhao C, Che D, Dickinson T, Wickham E, Bierle J, Doucet D, Milewski M, Yang R, Siegmund C, Haas J, Zhou L, Oliphant A, Fan JB, Barnard S, Chee MS (2004). Decoding randomly ordered DNA arrays. Genome Res.

[13] Kapranov P, Sementchenko VI, Gingeras TR (2003). Beyond expression profiling: next generation uses of high density oligonucleotide arrays. Brief Funct Genomic Proteomic.

[14] Mansfield ES, Sarwal MM (2004). Arraying the orchestration of allograft pathology. Am J Transplant.

[15] Golub TR, Slonim DK, Tamayo P, Huard C, Gaasenbeek M, Mesirov JP, Coller H, Loh ML, Downing JR, Caligiuri MA, Bloomfield CD, Lander ES (1999). Molecular classification of cancer: class discovery and class prediction by gene expression monitoring. Science.

[16] van’t Veer LJ, Dai H, van de Vijver MJ, He YD, Hart AA, Mao M, Peterse HL, van der Kooy K, Marton MJ, Witteveen AT, Schreiber GJ, Kerkhoven RM, Roberts C, Linsley PS, Bernards R, Friend SH (2002). Gene expression profiling predicts clinical outcome of breast cancer. Nature.

[17] Singh D, Febbo PG, Ross K, Jackson DG, Manola J, Ladd C, Tamayo P, Renshaw AA, D’Amico AV, Richie JP, Lander ES, Loda M, Kantoff PW, Golub TR, Sellers WR (2002). Gene expression correlates of clinical prostate cancer behavior. Cancer Cell.

[18] Wang T, Hopkins D, Schmidt C, Silva S, Houghton R, Takita H, Repasky E, Reed SG (2000). Identification of genes differentially over-expressed in lung squamous cell carcinoma using combination of cDNA subtraction and microarray analysis. Oncogene.

[19] Alon U, Barkai N, Notterman DA, Gish K, Ybarra S, Mack D, Levine AJ (1999). Broad patterns of gene expression revealed by clustering analysis of tumor and normal colon tissues probed by oligonucleotide arrays. Proc Natl Acad Sci USA.

[20] Ramaswamy S, Tamayo P, Rifkin R, Mukherjee S, Yeang CH, Angelo M, Ladd C, Reich M, Latulippe E, Mesirov JP, Poggio T, Gerald W, Loda M, Lander ES, Golub TR (2001). Multiclass cancer diagnosis using tumor gene expression signatures. Proc Natl Acad Sci USA.

[21] Brachat A, Pierrat B, Xynos A, Brecht K, Simonen M, Brungger A, Heim J (2002). A microarray-based, integrated approach to identify novel regulators of cancer drug response and apoptosis. Oncogene.

[22] Rhodes DR, Yu J, Shanker K, Deshpande N, Varambally R, Ghosh D, Barrette T, Pandey A, Chinnaiyan AM (2004). Large-scale meta-analysis of cancer microarray data identifies common transcriptional profiles of neoplastic transformation and progression. Proc Natl Acad Sci USA.

[23] Cutler DJ, Zwick ME, Carrasquillo MM, Yohn CT, Tobin KP, Kashuk C, Mathews DJ, Shah NA, Eichler EE, Warrington JA, Chakravarti A (2001). High-throughput variation detection and genotyping using microarrays. Genome Res.

[24] Yan PS, Chen CM, Shi H, Rahmatpanah F, Wei SH, Caldwell CW, Huang TH (2001). Dissecting complex epigenetic alterations in breast cancer using CpG island microarrays. Cancer Res.

[25] Pollack JR, Perou CM, Alizadeh AA, Eisen MB, Pergamenschikov A, Williams CF, Williams CF, Jeffrey SS, Botstein D, Brown PO (1999). Genome-wide analysis of DNA copy-number changes using cDNA microarrays. Nat Genet.

[26] Relogio A, Ben-Dov C, Baum M, Ruggiu M, Gemund C, Benes V, Darnell RB, Valcárcel J (2005). Alternative splicing microarrays reveal functional expression of neuron-specific regulators in Hodgkin lymphoma cells. J Biol Chem.

[27] Wang D, Coscoy L, Zylberberg M, Avila PC, Boushey HA, Ganem D, DeRisi JL (2002). Microarray-based detection and genotyping of viral pathogens. Proc Natl Acad Sci USA.

[28] Conejero-Goldberg C, Wang E, Yi C, Goldberg TE, Jones-Brando L, Marincola FM, Webster MJ, Torrey EF (2005). Infectious pathogen detection arrays: viral detection in cell lines and postmortem brain tissue. Biotechniques.

[29] Ji R, Cheng Y, Yue J, Yang J, Liu X, Chen H, Dean DB, Zhang C (2007). MicroRNA expression signature and antisense-mediated depletion reveal an essential role of microRNA in vascular neointimal lesion formation. Circ Res.

[30] Vasconcellos LM, Schachter AD, Zheng XX, Vasconcellos LH, Shapiro M, Harmon WE, Strom TB (1998). Cytotoxic lymphocyte gene expression in peripheral blood leukocytes correlates with rejecting renal allografts. Transplantation.

[31] Li B, Hartono C, Ding R, Sharma VK, Ramaswamy R, Qian B, Serur D, Mouradian J, Schwartz JE, Suthanthiran M (2001). Noninvasive diagnosis of renal-allograft rejection by measurement of messenger RNA for perforin and granzyme B in urine. N Engl J Med.

[32] Strehlau J, Pavlakis M, Lipman M, Shapiro M, Vasconcellos L, Harmon W, Strom TB (1997). Quantitative detection of immune activation transcripts as a diagnostic tool in kidney transplantation. Proc Natl Acad Sci USA.

[33] Lipman ML, Stevens AC, Strom TB (1994). Heightened intragraft CTL gene expression in acutely rejecting renal allografts. J Immunol.

[34] Halloran PF, Urmson J, Ramassar V, Melk A, Zhu LF, Halloran BP, Bleackley RC (2004). Lesions of T-cell-mediated kidney allograft rejection in mice do not require perforin or granzymes A and B. Am J Transplant.

[35] van de Vijver MJ, He YD, van’t Veer LJ, Dai H, Hart AA, Voskuil DW, Schreiber GJ, Peterse JL, Roberts C, Marton MJ, Parrish M, Atsma D, Witteveen A, Glas A, Delahaye L, van der Velde T, Bartelink H, Rodenhuis S, Rutgers ET, Friend SH, Bernards R (2002). A gene-expression signature as a predictor of survival in breast cancer. N Engl J Med.

[36] Alizadeh AA, Eisen MB, Davis RE, Ma C, Lossos IS, Rosenwald A, Boldrick JC, Sabet H, Tran T, Yu X, Powell JI, Yang L, Marti GE, Moore T, Hudson J, Lu L, Lewis DB, Tibshirani R, Sherlock G, Chan WC, Greiner TC, Weisenburger DD, Armitage JO, Warnke R, Levy R, Wilson W, Grever MR, Byrd JC, Botstein D, Brown PO, Staudt LM (2000). Distinct types of diffuse large B-cell lymphoma identified by gene expression profiling. Nature.

[37] Hauser P, Schwarz C, Mitterbauer C, Regele HM, Muhlbacher F, Mayer G, Mühlbacher F, Mayer G, Perco P, Mayer B, Meyer TW, Oberbauer R (2004). Genome-wide gene-expression patterns of donor kidney biopsies distinguish primary allograft function. Lab Invest.

[38] Serinsoz E, Bock O, Gwinner W, Schwarz A, Haller H, Kreipe H, Mengel M (2005). Local complement C3 expression is upregulated in humoral and cellular rejection of renal allografts. Am J Transplant.

[39] Sacks SH, Zhou W (2005). Allograft rejection: effect of local synthesis of complement. Springer Semin Immunopathol.

[40] Leslie JA, Meldrum KK (2008). The role of interleukin-18 in renal injury. J Surg Res.

[41] Tinckam KJ, Chandraker A (2006). Mechanisms and role of HLA and non-HLA alloantibodies. Clin J Am Soc Nephrol.

[42] Racusen LC, Haas M (2006). Antibody-mediated rejection in renal allografts: lessons from pathology. Clin J Am Soc Nephrol.

[43] Einecke G, Broderick G, Sis B, Halloran PF (2007). Early loss of renal transcripts in kidney allografts: relationship to the development of histologic lesions and alloimmune effector mechanisms. Am J Transplant.

[44] Li L, Ying L, Naesens M, Xiao W, Sigdel T, Hsieh S, Martin J, Chen R, Liu K, Mindrinos M, Davis R, Sarwal M (2007). Interference of globin genes with biomarker discovery for allograft rejection in peripheral blood samples. Physiol Genomics.

[45] Zhang Q, Reed EF (2006). Array-based methods for diagnosis and prevention of transplant rejection. Expert Rev Mol Diagn.

[46] Deng MC, Eisen HJ, Mehra MR, Billingham M, Marboe CC, Berry G, Kobashigawa J, Johnson FL, Starling RC, Murali S, Pauly DF, Baron H, Wohlgemuth JG, Woodward RN, Klingler TM, Walther D, Lal PG, Rosenberg S, Hunt S, CARGO Investigators (2006). Noninvasive discrimination of rejection in cardiac allograft recipients using gene expression profiling. Am J Transplant.

[47] Morgun A, Shulzhenko N, Perez-Diez A, Diniz RV, Sanson GF, Almeida DR, Matzinger P, Gerbase-DeLima M (2006). Molecular profiling improves diagnoses of rejection and infection in transplanted organs. Circ Res.

[48] Martinez-Llordella M, Puig-Pey I, Orlando G, Ramoni M, Tisone G, Rimola A, Lerut J, Latinne D, Margarit C, Bilbao I, Brouard S, Hernández-Fuentes M, Soulillou JP, Sánchez-Fueyo A (2007). Multiparameter immune profiling of operational tolerance in liver transplantation. Am J Transplant.

[49] Brouard S, Mansfield E, Braud C, Li L, Giral M, Hsieh SC, Baeten D, Zhang M, Ashton-Chess J, Braudeau C, Hsieh F, Dupont A, Pallier A, Moreau A, Louis S, Ruiz C, Salvatierra O, Soulillou JP, Sarwal M (2007). Identification of a peripheral blood transcriptional biomarker panel associated with operational renal allograft tolerance. Proc Natl Acad Sci USA.

[50] Gourishankar S, Halloran PF (2002). Late deterioration of organ transplants: a problem in injury and homeostasis. Curr Opin Immunol.

[51] Grigoryev DN, Liu M, Cheadle C, Barnes KC, Rabb H (2006). Genomic profiling of kidney ischemia-reperfusion reveals expression of specific alloimmunity-associated genes: Linking “immune” and “nonimmune” injury events. Transplant Proc.

[52] Park WD, Stegall MD (2007). A meta-analysis of kidney microarray datasets: investigation of cytokine gene detection and correlation with rt-PCR and detection thresholds. BMC Genomics.

[53] Eikmans M, Roos-van Groningen MC, Sijpkens YW, Ehrchen J, Roth J, Baelde HJ, Bajema IM, de Fijter JW, de Heer E, Bruijn JA (2005). Expression of surfactant protein-C, S100A8, S100A9, and B cell markers in renal allografts: investigation of the prognostic value. J Am Soc Nephrol.

[54] Kurian SM, Flechner SM, Kaouk J, Modlin C, Goldfarb D, Cook DJ, Head S, Salomon DR (2005). Laparoscopic donor nephrectomy gene expression profiling reveals upregulation of stress and ischemia associated genes compared to control kidneys. Transplantation.

[55] Hotchkiss H, Chu TT, Hancock WW, Schroppel B, Kretzler M, Schmid H, Liu Y, Dikman S, Akalin E (2006). Differential expression of profibrotic and growth factors in chronic allograft nephropathy. Transplantation.

[56] Flechner SM, Kurian SM, Head SR, Sharp SM, Whisenant TC, Zhang J, Chismar JD, Horvath S, Mondala T, Gilmartin T, Cook DJ, Kay SA, Walker JR, Salomon DR (2004). Kidney transplant rejection and tissue injury by gene profiling of biopsies and peripheral blood lymphocytes. Am J Transplant.

[57] Donauer J, Rumberger B, Klein M, Faller D, Wilpert J, Sparna T, Schieren G, Rohrbach R, Dern P, Timmer J, Pisarski P, Kirste G, Walz G (2003). Expression profiling on chronically rejected transplant kidneys. Transplantation.

[58] Scherer A, Krause A, Walker JR, Korn A, Niese D, Raulf F (2003). Early prognosis of the development of renal chronic allograft rejection by gene expression profiling of human protocol biopsies. Transplantation.

[59] Michiels S, Koscielny S, Hill C (2005). Prediction of cancer outcome with microarrays: a multiple random validation strategy. Lancet.

[60] Famulski KS, Einecke G, Reeve J, Ramassar V, Allanach K, Mueller T, Hidalgo LG, Zhu LF, Halloran PF (2006). Changes in the transcriptome in allograft rejection: IFN-gamma-induced transcripts in mouse kidney allografts. Am J Transplant.

[61] Einecke G, Melk A, Ramassar V, Zhu LF, Bleackley RC, Famulski KS, Halloran PF (2005). Expression of CTL associated transcripts precedes the development of tubulitis in T-cell mediated kidney graft rejection. Am J Transplant.

[62] Melk A, Mansfield ES, Hsieh SC, Hernandez-Boussard T, Grimm P, Rayner DC, Halloran PF, Sarwal MM (2005). Transcriptional analysis of the molecular basis of human kidney aging using cDNA microarray profiling. Kidney Int.

[63] Brazma A, Vilo J (2000). Gene expression data analysis. FEBS Lett.

[64] Azuaje F, Dopazo J (2005). Data analysis and visualization in genomics and proteomics.

[65] Bird AP (1986). CpG-rich islands and the function of DNA methylation. Nature.

[66] Henikoff S, Matzke MA (1997). Exploring and explaining epigenetic effects. Trends Genet.

[67] Laird PW (2003). The power and the promise of DNA methylation markers. Nat Rev Cancer.

[68] Grewal A, Conway A (2000). Tools for analyzing microarray expression data. J Lab Autom.

[69] Schumacher A, Kapranov P, Kaminsky Z, Flanagan J, Assadzadeh A, Yau P, Virtanen C, Winegarden N, Cheng J, Gingeras T, Petronis A (2006). Microarray-based DNA methylation profiling: technology and applications. Nucleic Acids Res.

[70] Chang HY, Nuyten DS, Sneddon JB, Hastie T, Tibshirani R, Sorlie T, Dai H, He YD, van’t Veer LJ, Bartelink H, van de Rijn M, Brown PO, van de Vijver MJ (2005). Robustness, scalability, and integration of a wound-response gene expression signature in predicting breast cancer survival. Proc Natl Acad Sci USA.

[71] Getz G, Levine E, Domany E (2000). Coupled two-way clustering analysis of gene microarray data. Proc Natl Acad Sci USA.

[72] Wu J, Smith LT, Plass C, Huang TH (2006). ChIP-chip comes of age for genome-wide functional analysis. Cancer Res.

[73] Beyer A, Workman C, Hollunder J, Radke D, Moller U, Wilhelm T, Ideker T (2006). Integrated assessment and prediction of transcription factor binding. PLoS Comput Biol.

[74] Gardner L (2002). The c-Myc oncogenic transcription factor.

[75] Shi L, Reid LH, Jones WD, Shippy R, Warrington JA, Baker SC, Collins PJ, de Longueville F, Kawasaki ES, Lee KY, Luo Y, Sun YA, Willey JC, Setterquist RA, Fischer GM, Tong W, Dragan YP, Dix DJ, Frueh FW, Goodsaid FM, Herman D, Jensen RV, Johnson CD, Lobenhofer EK, Puri RK, Schrf U, Thierry-Mieg J, Wang C, Wilson M, Wolber PK, Zhang L, Amur S, Bao W, Barbacioru CC, Lucas AB, Bertholet V, Boysen C, Bromley B, Brown D, Brunner A, Canales R, Cao XM, Cebula TA, Chen JJ, Cheng J, Chu TM, Chudin E, Corson J, Corton JC, Croner LJ, Davies C, Davison TS, Delenstarr G, Deng X, Dorris D, Eklund AC, Fan XH, Fang H, Fulmer-Smentek S, Fuscoe JC, Gallagher K, Ge W, Guo L, Guo X, Hager J, Haje PK, Han J, Han T, Harbottle HC, Harris SC, Hatchwell E, Hauser CA, Hester S, Hong H, Hurban P, Jackson SA, Ji H, Knight CR, Kuo WP, LeClerc JE, Levy S, Li QZ, Liu C, Liu Y, Lombardi MJ, Ma Y, Magnuson SR, Maqsodi B, McDaniel T, Mei N, Myklebost O, Ning B, Novoradovskaya N, Orr MS, Osborn TW, Papallo A, Patterson TA, Perkins RG, Peters EH, Peterson R, Philips KL, Pine PS, Pusztai L, Qian F, Ren H, Rosen M, Rosenzweig BA, Samaha RR, Schena M, Schroth GP, Shchegrova S, Smith DD, Staedtler F, Su Z, Sun H, Szallasi Z, Tezak Z, Thierry-Mieg D, Thompson KL, Tikhonova I, Turpaz Y, Vallanat B, Van C, Walker SJ, Wang SJ, Wang Y, Wolfinger R, Wong A, Wu J, Xiao C, Xie Q, Xu J, Yang W, Zhang L, Zhong S, Zong Y, Slikker W (2006). The MicroArray Quality Control (MAQC) project shows inter- and intraplatform reproducibility of gene expression measurements. Nat Biotechnol.

[76] Higgins JP, Wang L, Kambham N, Montgomery K, Mason V, Vogelmann SU, Lemley KV, Brown PO, Brooks JD, van de Rijn M (2004). Gene expression in the normal adult human kidney assessed by complementary DNA microarray. Mol Biol Cell.

[77] Ramaswamy S, Perou CM (2003). DNA microarrays in breast cancer: the promise of personalised medicine. Lancet.

[78] Russo G, Zegar C, Giordano A (2003). Advantages and limitations of microarray technology in human cancer. Oncogene.

[79] Gimino VJ, Lande JD, Berryman TR, King RA, Hertz MI (2003). Gene expression profiling of bronchoalveolar lavage cells in acute lung rejection. Am J Respir Crit Care Med.

[80] Brazma A, Hingamp P, Quackenbush J, Sherlock G, Spellman P, Stoeckert C, Aach J, Ansorge W, Ball CA, Causton HC, Gaasterland T, Glenisson P, Holstege FC, Kim IF, Markowitz V, Matese JC, Parkinson H, Robinson A, Sarkans U, Schulze-Kremer S, Stewart J, Taylor R, Vilo J, Vingron M (2001). Minimum information about a microarray experiment (MIAME)—toward standards for microarray data. Nat Genet.

[81] Spellman PT, Miller M, Stewart J, Troup C, Sarkans U, Chervitz S, Bernhart D, Sherlock G, Ball C, Lepage M, Swiatek M, Marks WL, Goncalves J, Markel S, Iordan D, Shojatalab M, Pizarro A, White J, Hubley R, Deutsch E, Senger M, Aronow BJ, Robinson A, Bassett D, Stoeckert CJ Jr, Brazma A (2002) Design and implementation of microarray gene expression markup language (MAGE-ML). Genome Biol 3:RESEARCH0046 10.1186/gb-2002-3-9-research0046 PMC126871 12225585

[82] Perez-Iratxeta C, Andrade MA (2005). Inconsistencies over time in 5% of NetAffx probe-to-gene annotations. BMC Bioinformatics.

[83] Dai M, Wang P, Boyd AD, Kostov G, Athey B, Jones EG, Bunney WE, Myers RM, Speed TP, Akil H, Watson SJ, Meng F (2005). Evolving gene/transcript definitions significantly alter the interpretation of GeneChip data. Nucleic Acids Res.

[84] Harbig J, Sprinkle R, Enkemann SA (2005). A sequence-based identification of the genes detected by probesets on the Affymetrix U133 plus 2.0 array. Nucleic Acids Res.

[85] Chen R, Li L, Butte A (2007). AILUN: reannotating gene expression data automatically. Nat Methods.

[86] Shedden K, Chen W, Kuick R, Ghosh D, Macdonald J, Cho KR, Giordano TJ, Gruber SB, Fearon ER, Taylor JM, Hanash S (2005). Comparison of seven methods for producing Affymetrix expression scores based on False Discovery Rates in disease profiling data. BMC Bioinformatics.

[87] Mayo MS, Gajewski BJ, Morris JS (2006). Some statistical issues in microarray gene expression data. Radiat Res.

[88] Suarez-Farinas M, Magnasco MO (2007). Comparing microarray studies. Methods Mol Biol.

[89] Nagarajan S, Mansfield E, Hsieh S, Liu R, Hsieh F, Li L, Salvatierra O, Sarwal MM (2007). Transplant reno-vascular stenoses associated with early erythropoietin use. Clin Transplant.

[90] Park W, Griffin M, Grande JP, Cosio F, Stegall MD (2007). Molecular evidence of injury and inflammation in normal and fibrotic renal allografts one year posttransplant. Transplantation.

[91] Mas V, Maluf D, Archer K, Yanek K, Mas L, King A, Gibney E, Massey D, Cotterell A, Fisher R, Posner M (2007). Establishing the molecular pathway involved in chronic allograft nephropathy for testing new noninvasive diagnostic markers. Transplantation.

[92] Zhang HQ, Lu H, Enosawa S, Suzuki S, Takahara S, Nakajima T, Saito H, Sakamoto K (2004). Comprehensive examination of gene expression associated with long-term stable graft acceptance by renal transplant recipients. Clin Transplant.

[93] Kainz A, Mitterbauer C, Hauser P, Schwarz C, Regele HM, Berlakovich G, Mayer G, Perco P, Meyer B, Meyer TW, Oberbauer R (2004). Alterations in gene expression in cadaveric vs. live donor kidneys suggest impaired tubular counterbalance of oxidative stress at implantation. Am J Transplant.

[94] Flechner SM, Kurian SM, Solez K, Cook DJ, Burke JT, Rollin H, Hammond JA, Whisenant T, Lanigan CM, Head SR, Salomon DR (2004). De novo kidney transplantation without use of calcineurin inhibitors preserves renal structure and function at two years. Am J Transplant.

[95] Chua MS, Barry C, Chen X, Salvatierra O, Sarwal MM (2003). Molecular profiling of anemia in acute renal allograft rejection using DNA microarrays. Am J Transplant.

[96] Zhang HQ, Lu H, Enosawa S, Takahara S, Sakamoto K, Nakajima T, Saito H, Suzuki S (2002). Microarray analysis of gene expression in peripheral blood mononuclear cells derived from long-surviving renal recipients. Transplant Proc.

[97] Akalin E, Hendrix RC, Polavarapu RG, Pearson TC, Neylan JF, Larsen CP, Lakkis FG (2001). Gene expression analysis in human renal allograft biopsy samples using high-density oligoarray technology. Transplantation.

[98] Kusaka M, Kuroyanagi Y, Kowa H, Nagaoka K, Mori T, Yamada K, Shiroki R, Kurahashi H, Hoshinaga K (2007). Genomewide expression profiles of rat model renal isografts from brain dead donors. Transplantation.

[99] Berthier CC, Lods N, Joosten SA, Kooten C, Leppert D, Lindberg RL, Kappeler A, Raulf F, Sterchi EE, Lottaz D, Marti HP (2006). Differential regulation of metzincins in experimental chronic renal allograft rejection: potential markers and novel therapeutic targets. Kidney Int.

[100] Djamali A, Reese S, Oberley T, Hullett D, Becker B (2005). Heat shock protein 27 in chronic allograft nephropathy: a local stress response. Transplantation.

[101] Schuurs TA, Gerbens F, Hoeven JA, Ottens PJ, Kooi KA, Leuvenink HG, Hofstra RM, Ploeg RJ (2004). Distinct transcriptional changes in donor kidneys upon brain death induction in rats: insights in the processes of brain death. Am J Transplant.

[102] Leonard MO, Kieran NE, Howell K, Burne MJ, Varadarajan R, Dhakshinamoorthy S, Porter AG, O’Farrelly C, Rabb H, Taylor CT (2006). Reoxygenation-specific activation of the antioxidant transcription factor Nrf2 mediates cytoprotective gene expression in ischemia-reperfusion injury. FASEB J.

